# Ultrasonography has a diagnostic value in the assessment of cervical radiculopathy: A prospective pilot study

**DOI:** 10.1007/s00330-016-4704-9

**Published:** 2017-01-03

**Authors:** Mikinobu Takeuchi, Norimitsu Wakao, Atsuhiko Hirasawa, Kenta Murotani, Mitsuhiro Kamiya, Koji Osuka, Masakazu Takayasu

**Affiliations:** 1 Spine Center, Aichi Medical University Aichi Medical University, Nagakute, Aichi Japan; 2 Department of Neurological Surgery, Aichi Medical University Aichi Medical University, Karimata 1-1 Yazako, Nagakute City, Aichi Prefecture Japan; 3 Department of Orthopedic Surgery, Aichi Medical University Aichi Medical University, Nagakute, Aichi Japan; 4 Department of Biostatistics and Clinical Research Center, Aichi Medical University Aichi Medical University, Nagakute, Aichi Japan

**Keywords:** Ultrasound, Cervical nerve root, Cervical radiculopathy, Ultrasonography, Cross-sectional area

## Abstract

**Objective:**

This study investigated the diagnostic accuracy of the difference in the cross-sectional areas (CSAs) of affected cervical nerve roots (NRs) for diagnosing cervical radiculopathy (CR).

**Methods:**

In total, 102 CR patients and 219 healthy volunteers were examined with ultrasound. The CSA of the cervical NR at each level was measured on the affected side and the contralateral side in CR patients by blinded ultrasonographic technicians. The difference between the CSAs of CR patients and normal volunteers and the difference in the laterality of CSA at the same affected level (ΔCSA) were calculated for each cervical level.

**Results:**

The CSAs of the affected NRs in CR patients were significantly larger than those of the unaffected NRs in CR patients and those of the control group at the C5, C6 and C7 levels (P<0.005). ΔCSA was also significantly larger in the CR group at all levels (P<0.001). A receiver operating characteristic analysis demonstrated that the threshold values were 9.6 mm^2^ (CSA) for C5NR and 15 mm^2^ for both C6NR and C7NR.

**Conclusions:**

This study revealed that the CSAs of affected NRs were enlarged and that the laterality of the CSA (ΔCSA) was greater in CR patients than in control patients.

***Key Points*:**

• *Cervical radiculopathy is diagnosed through ultrasonographic measurement of the CSAs*.

• *The CSAs of affected nerve roots were significantly enlarged*.

• *The ΔCSA in the CR group was significantly higher than in the control group*.

• *Diagnostic CSA and ΔCSA thresholds were identified*.

## Introduction

Magnetic resonance imaging (MRI), which can be performed either with or without computerized tomography myelography (CTM), is considered the best diagnostic technique for cervical radiculopathy (CR) [1]. However, MRI is expensive, and it is not readily available. MRI is also contraindicated in patients with a pacemaker, defibrillator or deep brain stimulator. Therefore, MRI is not always appropriate for routine screening. In contrast, ultrasonography provides higher spatial-resolution images that are easily and rapidly obtained in any environment, which makes it suitable for screening. Some recent studies have used cervical nerve root (NR) ultrasonography for the ultrasonographic diagnosis of chronic inflammatory demyelinating polyradiculoneuropathy [2, 3] and amyotrophic lateral sclerosis [4], and to measure the normal cross-sectional area (CSA) of cervical NRs [5, 6]. These studies aided in the development of cervical NR ultrasonography.

Some ultrasonographic studies have reported that nerve CSA enlargement exhibited high diagnostic sensitivity and specificity for the study of focal nerve damage in cases of compression, trauma and entrapment syndromes [7–9]. MRI often shows multilevel compression; In addition, the degree of nerve swelling distal to the compression in its extraspinal segment as assessed by ultrasonography may better indicate which NR is most affected.

We hypothesized that NRs compressed by cervical disc herniation or cervical osteophytes would exhibit swelling and that the swollen cervical NRs would be detectable using ultrasonography. This prospective study evaluated the accuracy of ultrasound for the diagnosis of CR by comparing the CSAs of cervical NRs in patients with those of normal volunteers, and the differences between the affected and unaffected CSAs at the same cervical level were examined. In the strict anatomical sense, it should be noted that ultrasound measures the large anterior branch of the spinal nerve of the respective cervical level rather than the cervical NR.

## Materials and methods

### Patients

The institutional review board approved this study, and informed consent was obtained from all subjects. This prospective pilot cohort study was performed between January 2013 and November 2015 at our hospital and included patients with CR.

Inclusion criteria were: (1) Patients were required to exhibit CR symptoms, such as arm, scapular or periscapular pain; paraesthesias; numbness and sensory changes; weakness; or abnormal deep tendon reflexes in the arm. (2) Cervical MRI was required to reveal cervical disc herniation or cervical osteophytes that compressed cervical NRs. Four senior spinal surgeons used cervical MRI as the reference standard to definitively diagnose CR prior to cervical NR ultrasonography.

Patients were excluded if they had any of the following conditions: (1) myelopathy; (2) neck pain only; (3) severe diabetes or renal or liver dysfunction; (4) myopathy, neuropathy or collagen diseases; (5) no signs of root compression on cervical MRI, even if the patients had neurological defects; (6) bilateral CR; and (7) trauma.

The control group consisted of 219 healthy volunteers with no clinical signs or symptoms of CR. These same individuals were also included in a previous study [10] that analysed the CSA of cervical NRs in normal subjects. In contrast, in this manuscript, we report the CSAs in CR patients compared with the CSAs in 219 normal subjects.

### Ultrasonographic measurements

Three ultrasonographic technicians with 5 years of experience in musculoskeletal ultrasonographic examination and who were unaware of the patients’ histories and clinical and MRI results performed ultrasonography using a real-time scanner with a 13-5 MHz linear array probe within 2 weeks of the cervical MRI.

All patients were placed lying down in a lateral position as described previously [10]. The examiner set the linear array probe of the ultrasound machine to the brightness mode while standing behind the subject. We identified each NR using axial methods. The examiners identified the C5 to C7 NRs (C5NR, C6NR and C7NR) in an axial image according to the shapes of the transverse processes. The C5 and C6 transverse processes have obvious anterior and posterior tubercles, and the C7 transverse process has a rudimentary anterior tubercle and a prominent posterior tubercle. Therefore, the C7 vertebral level is a key landmark [11].

The examiners measured the diameter (D, mm) and transverse diameter (TD, mm) and calculated the cross-sectional area (CSA, mm^2^ = D×TD×π/4) of bilateral C5NR, C6NR and C7NR.

**Fig. 1:**
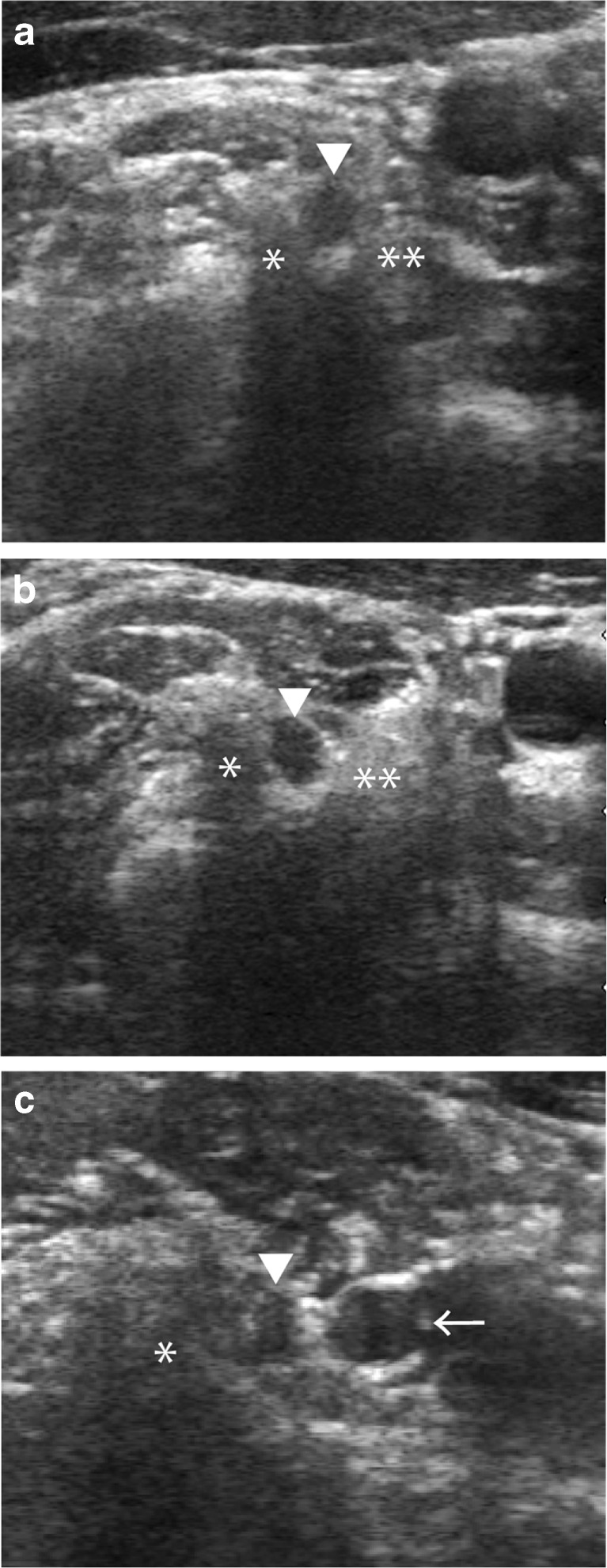
Axial ultrasonographic images (**a**: C5, **b**: C6, **c**: C7 level) of the cervical NRs of a normal subject. (**a**) Axial ultrasonographic image showing the C5 (▼) NR between the anterior (**) and posterior processes (*) at the C5 level. (**b**) Axial ultrasonographic image showing the C6 (▼) NR between the anterior (**) and posterior processes (*) at the C6 level. (**c**) Axial ultrasonographic image showing the C7 (▼) NR, vertebral artery (←) and posterior process (*) at the C7 level. *NR* nerve root

We standardized the measurement location of each NR in this study. The D and TD of C5NR and C6NR were measured, and their CSAs were calculated where the NRs just appeared between the anterior and posterior tubercles of the transverse process, which is the lateral zone [12] (Fig. 1 a, b). We measured The D and TD of C7NRs where C7NR presented at the same level as the posterior tubercle of the C7 level (Fig. 1 c).

We assessed the following four components:

1. The CSAs of C5NR, C6NR and C7NR of subjects with CR (CR group) were compared with those of previously reported normal volunteers (control group) [10] at the same cervical nerve level.
2. The value of the difference between the affected and unaffected CSA was calculated at the same cervical nerve level in CR patients.
3. The difference in the laterality of CSA (ΔCSA mm^2^) between the CR group and the control group was calculated at each cervical nerve level.
4. The sensitivity, specificity and cut-off values of the CSA and ΔCSA for C5NR, C6NR and C7NR were examined.

### Statistical analysis

After testing the normal distribution using the Shapiro-Wilk test, the CSAs of C5NR, C6NR and C7NR in the CR patients and normal volunteers were compared using a non-parametric test (Mann-Whitney U test). The CSAs between the affected and unaffected side of C5NR, C6NR and C7NR in the CR patients were compared using a Wilcoxon signed-rank test. The difference in the ΔCSA mm^2^ between the CR group and the control group at each cervical level was compared using a Mann-Whitney U test. The receiver operating characteristic (ROC) curve was used to examine the sensitivity, specificity and cut-off values of the CSA and ΔCSA for C5NR, C6NR and C7NR. We analysed age and sex differences between the patients and normal volunteers using the chi-square test or Mann-Whitney U test. The results are expressed as the median and interquartile range (IQR). To assess the reliability of ultrasonographic measurements, four examiners investigated the CSAs at C5NR, C6NR and C7NR in the same five normal volunteers to determine the inter- and intra-observer reliability. The data were analysed using SPSS software (version 22; SPSS, Chicago, IL, USA). The significance level was set to 0.05.

## Results

In the CR group, 65 patients were male and 37 patients were female, with a mean age of 52±12 years (range 28–82). CRs for C5NR, C6NR or C7NR were diagnosed in 10, 48 and 44 patients, respectively. A total of 102 cervical NRs were affected.

The control group comprised 219 healthy volunteers (99 males, 120 females) with an overall mean age of 49±15 years (range 20–79 years) [10]. There was a significant difference between the two groups with regard to sex (P=0.001, chi-square test) but not age (P=0.51, Mann-Whitney U test).

**Fig. 2:**
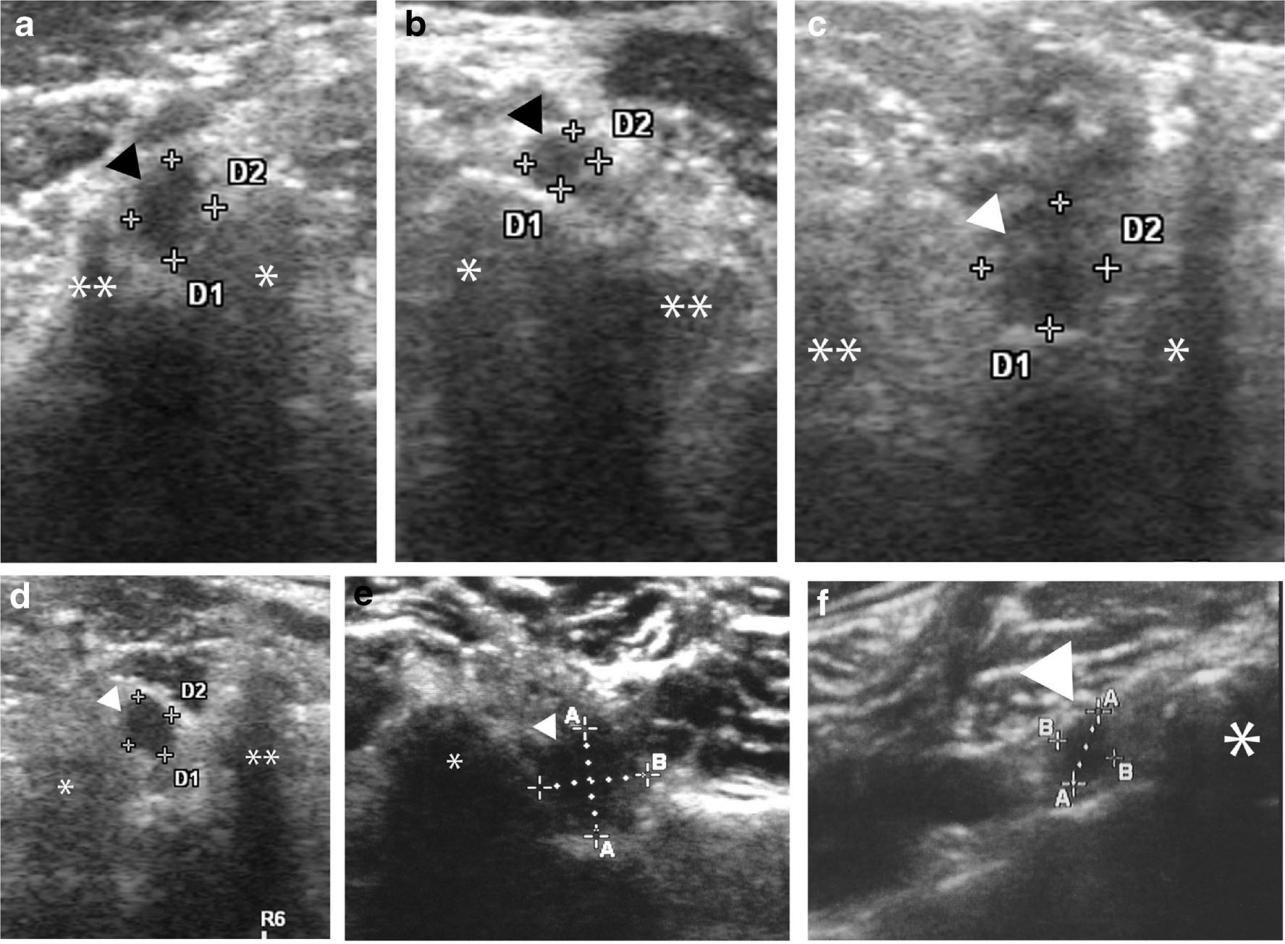
Axial ultrasonographic images. (**a**) Affected side at C5, (**b**) unaffected side at C5, (**c**) affected side at C6, (**d**) unaffected side at C6, (**e**) affected side at C7, (**f**) unaffected side at C7 level, of cervical NRs in a patient with cervical radiculopathy. ▼ cervical NR, * posterior process, ** anterior process, *D* nerve root diameter (mm), *TD* nerve root transverse diameter, *NR* nerve root. CSA = D×TD×π/4 (mm^2^)

The median CSAs were 6.3 (C5NR), 10.7 (C6NR) and 8.8 mm^2^ (C7NR) in the control group. In the CR group, the median CSAs of the unaffected side were 7.1, 12.2 and 10.5 mm^2^, and those of the affected side were 14.5, 20.4 and 18.8 mm^2^. The median CSAs of C5NR, C6NR and C7NR were significantly larger in the CR group than in the control group (P<0.001) (Table 1 and Fig. 2). The median CSAs of C5NR, C6NR and C7NR were significantly larger on the affected side than on the unaffected side in the CR group (P<0.001). There were no significant differences in CSAs between the control group and the unaffected side of the CR group (C5: P=0.52, C6: P=0.11, C7: P=0.06) (Table 1 and Fig. 2). The median ΔCSAs were 1.2 (C5NR), 1.8 (C6NR) and 1.4 mm^2^ (C7NR) in the control group. The median ΔCSAs in the CR group were 6.5, 8.9 and 8.0 mm^2^. There was a significant difference in the ΔCSAs of C5NR, C6NR and C7NR between the control and the CR groups (P<0.001) (Table 2).

**Table 1:** Nerve measurements in the control group and the CR group

	CSA		
	C5NR	C6NR	C7NR
Control	6.3 mm^2^	10.7	8.8
IQR	(5.1–7.5)	(8.8–13.2)	(7.2–11.3)
CR group			
Unaffected side	7.1	12.2	10.4
IQR	(5.1–9.7)	(10.0–13.9)	(8.3–11.3)
Affected side	14.5	20.4	18.8
IQR	(14.1–16.0)	(18.0–22.5)	(16.2–20.9)
No. of patients	10	48	44
P-value (Control vs. Affected side)	<0.001	<0.001	<0.001
P-value (Unaffected vs. Affected side)	<0.001	<0.001	<0.01
P-value (Control vs. Unaffected side)	0.52	0.11	0.06

*Note*. Unless otherwise indicated, the data are medians, with the inter-quartile range (IQR) in parentheses

*CR* cervical radiculopathy, NR nerve root, CSA cross-sectional area

**Table 2:** Laterality in the control group and the CR group

	ΔCSA		
	C5NR	C6NR	C7NR
Control	1.2 mm^2^	1.8	1.4
IQR	(0.5–2.5)	(0.8–3.3)	(0.8–3.0)
CR group	6.5	8.9	8.0
IQR	(4.5–8.9)	(4.2–10.9)	(5.4–13.4)
No. of patients	10	44	48
P-value	<0.001	<0.001	<0.001

*Note*. Unless otherwise indicated, the data are medians, with the inter-quartile range (IQR) in parentheses

*CR* cervical radiculopathy, *NR* nerve root, *ΔCSA* laterality of the cross-sectional area

Table 3 presents the threshold values of the CSA and ΔCSA obtained using ROC analysis. The threshold values for C5NR were 9.6 mm^2^ (CSA), with 90% sensitivity and 91% specificity, and 4.0 (ΔCSA), with 90% sensitivity and 93% specificity. The threshold values for C6NR were 15 mm^2^ (CSA), with 92% sensitivity and 90% specificity, and 7 (ΔCSA), with 69% sensitivity and 98% specificity. The threshold values for C7NR were 15 mm^2^ (CSA), with 89% sensitivity and 96% specificity, and 5 (ΔCSA), with 80% sensitivity and 94% specificity. The areas under the curve (AUCs) for CSA were greater than those of ΔCSA for C5NR, C6NR and C7NR. There were significant differences for C6NR between CSA (AUC: 0.98 [95% CI 0.96–0.99]) and ΔCSA (AUC: 0.89 [95% CI 0.84–0.95]) (P<0.001). However, there were no significant differences between CSA and ΔCSA for C5NR or C7NR (P=0.3, and 0.07, respectively).

**Table 3:** Sensitivity and specificity of nerve measurements for the diagnosis of CR

Threshold	Sensitivity (%)^§^	Specificity (%)^§^	FP	FN	LR+	LR-	AUC^§^	P-value
C5NR-CSA							0.97	
8.5 mm^2^	100 (96.4–100)	83 (77.5–87.8)	17	0	5.9	0	(0.94–1)	
***9.6***	***90 (82.7–95.2)***	***91 (86.2–94.3)***	***9***	***10***	***10***	***0.11***		
13.6	80 (71.3–87.6)	100 (98.3–100)	0	20		0.2		
C5NR-ΔCSA							0.91	0.3
0.6	100 (96.4–100)	30 (24.1–36.9)	70	0	1.4	0	(0.79–1)	
***4.0***	***90 (82.7–95.2)***	***93 (89.0–96.1)***	***7***	***10***	***12.9***	***0.11***		
7.3	50 (94.7–100)	100 (98.3–100)	0	50		0.5		
C6NR-CSA							0.98	
12.6	100 (96.4–100)	72 (65.7–78.0))	28	0	3.6	0	(0.96–0.99)	
***15***	***92 (85.1–96.6)***	***90 (85.2–93.6)***	***10***	***8***	***9.2***	***0.09***		
17	79 (70.3–86.8)	98 (95.4–99.5)	2	21	39.5	0.21		
19	60 (49.6–69.4)	100 (98.3–100)	0	40		0.4		
C6NR-ΔCSA							0.89	0.001
2.2	100 (96.4–100)	30 (24.1–36.7)	70	0	1.4	0	(0.84–0.95)	
3	81 (72.4–88.4)	74 (67.6–79.7)	26	19	3.1	0.26		
***7***	***69 (58.7–77.5)***	***98 (95.4–99.5)***	***2***	***31***	***34.5***	***0.32***		
11.3	25 (17.4–35.1)	100 (98.3–100)	0	75		0.75		
C7NR-CSA							0.97	
10	100 (96.4–100)	63 (56.2–69.4)	37	0	2.7	0	(0.95–0.99)	
***15***	***89 (81.5–94.5)***	***96 (92.3–98.1)***	***4***	***11***	***22.3***	***0.11***		
21	25 (17.4–35.1)	100 (98.3–100)	0	75		0.75		
C7NR-ΔCSA							0.93	0.07
1.7	98 (93.1–99.8)	57 (50.2–63.7)	43	2	2.3	0.04	(0.89–0.97)	
***5***	***80*** **(71.4–878.6)**	***94 (90.1–96.8)***	***6***	***20***	***13.3***	***0.21***		
12	32 (23.4–42.3)	100 (98.3–100)	0	68		0.68		

^§^The data in parentheses are 95% confidence intervals

^¶^The data are the differences in AUCs between CSA and ΔCSA

*CR* cervical radiculopathy, *NR* nerve root, *CSA* cross-sectional area, *ΔCSA* laterality of the cross-sectional area, *FP* false positive, *FN* false negative, *LR+* positive likelihood ratio, *LR−* negative likelihood ratio

The bold type indicating the better threshold value

The inter-observer reliability of the CSA measurements (mm^2^) was 0.71 [95% CI 0.49–0.85]. The intra-observer reliability was 0.74 [95% CI 0.4–0.89], 0.71 [95% CI 0.37–0.87], 0.71 [95% CI 0.33–0.87] and 0.61 [95% CI 0.1–0.83] for the four examiners, respectively.

## Discussion

This prospective cohort study investigated the accuracy of an ultrasonographic diagnostic approach for CR detection. This goal was accomplished by measuring the CSAs of cervical NRs in 102 patients with cervical disc herniation or osteophyte compression and the comparison of these CSAs with those of 219 normal subjects. We obtained three important findings in this study. First, we determined that the threshold values of the CSA and the laterality of CSA (ΔCSA) for CR were 9.6 mm^2^ (CSA) and 4.0 (ΔCSA) for C5NR, 15 mm^2^ and 7 for C6NR, and 15 mm^2^ and 5 for C7NR. Second, the CSAs of affected NRs in the CR group were significantly larger than both the CSAs of NRs in the control group and the CSAs of unaffected NRs in the CR group. Third, the ΔCSA at the affected level was also significantly larger in the CR group than in the control group. We also demonstrated that the diagnostic ability of the measurement of CSA was superior to that of ΔCSA.

Peripheral nerve enlargement occurs in various entrapment neuropathies [13], for example carpal tunnel syndrome [14] and ulnar neuropathy at the elbow [15]. Studies have described the nerve enlargement proximal to the site of constriction. However, Simon et al. [16] reported that the enlargement of an entrapped peripheral nerve occurs at both the proximal and distal sides. The CSAs of the affected cervical NRs distal to the site of the compression lesion were also greater in patients with CR, as determined in the present cervical ultrasonographic study. Bianchi et al. [17] demonstrated that external compression of a peripheral nerve causes internal structural changes in entrapment diseases and that exacerbated venous perfusion causes increased intraneural interstitial pressure and reversible intraneural edema. Rao [18] also reported that intrinsic blood vessels in entrapment neuropathies exhibited increased permeability within the compressed NR, which secondarily results in chronic edema and fibrosis of the NR. There are modest negative correlations between the electrodiagnostic parameters (e.g. motor velocity across the elbow, compound muscle action potential (CMAP), amplitude, distal sensory nerve action potential (SNAP)) and the sonographic peripheral nerve diameter [19]. This finding suggests that loss of sensory and motor axons is associated with nerve enlargement. Therefore, we confirmed that measurement of the CSAs of cervical NRs using ultrasound can serve as a simple diagnostic tool for CR.

ΔCSA will also likely be useful as another diagnostic evaluation tool for CR. We demonstrated that the ΔCSA values were 6.5 (C5NR), 8.9 (C6NR) and 8.0 mm^2^ (C7NR) in the CR group, which were significantly larger than the values of 1.2, 1.8 and 1.4 mm^2^ in the control group. Additionally, we found larger CSAs on the affected side than on the unaffected side in the CR group. In contrast, Kim et al. [20] reported that the CSAs of specific NRs (C5NR, C6NR and C7NR) appeared larger on the affected side than on the unaffected side, but no significant differences were noted at any individual level. Study differences involving two aspects contributed to the difference between Kim’s results and ours: the number of patients and the inclusion criteria. First, Kim’s study assessed 24 patients (C5:5, C6:12 and C7:7), which was less than the 102 patients (C5:10, C6:48 and C7:44) in our study. Second, Kim’s report included patients with neck pain only, whereas we excluded patients with neck pain who otherwise lacked root compressive signs on the cervical MRI, even if the patients had neurological defects. Therefore, our study provides more accurate and rigorous results.

Comparison of the CSA and ΔCSA values using the AUC in an ROC analysis revealed that the diagnostic precision was almost identical for C5NR and C7NR. The AUC for the CSA of C6NR was significantly greater than that for ΔCSA. There are no articles regarding CR that precisely compare CSA or ΔCSA between patients and normal subjects. Therefore, we recommend that comparisons of the CSAs between affected and unaffected NRs at the same cervical level be conducted.

This study has some limitations. First, CRs at the C5 NR level were limited, and we excluded patients with CR at the C8 NR because the C8 NR does not have a distinctive landmark to standardize the measurement point. Second, the relationship between the measurement points of the cervical NR and dorsal root ganglia (DRG) of each cervical NRs was not clear. Yabuki et al. [21] reported that the incidence of a distal position of the DRG was 67% for the C6 NR and 50% for the C7 NR. The ultrasonographic measurement points in our study were far more distal than Yabuki’s measurement points because we measured the cervical NR using the transverse process, which is the lateral zone. Therefore, the DRG was barely included in this study. Third, there was a significant sex difference between the patient and control groups. However, this difference probably did not affect our results because our previous study [10] demonstrated that the CSA is not related to sex.

In conclusion, the diagnostic CSA and ΔCSA thresholds with high sensitivity and specificity for patients with CR were identified. We recommend measurement of the CSAs of affected and unaffected CRs at the same cervical level in order to increase the diagnostic accuracy of CR. These findings are important because CR due to cervical disc herniation or cervical osteophytes may be diagnosed rapidly and easily in any patient using our cervical ultrasonographic method.

## Abbreviations

ΔCSA: Laterality of the CSA
AUC: Area under the curve
CR: Cervical radiculopathy
CSA: Cross-sectional area
NR: Nerve root
ROC: Receiver operating characteristic
